# Nano drug delivery systems in upper gastrointestinal cancer therapy

**DOI:** 10.1186/s40580-020-00247-2

**Published:** 2020-12-10

**Authors:** Julia Salapa, Allison Bushman, Kevin Lowe, Joseph Irudayaraj

**Affiliations:** 1 Department of Bioengineering, University of Illinois at Urbana-Champaign, Urbana, IL 61801 USA; 2 Department of Physics, Technical University of Vienna, Karlsplatz 13, 1040 Vienna, Austria; 3 Department of Mechanical Science and Engineering, University of Illinois at Urbana-Champaign, Urbana, IL 61801 USA; 4 Carle Foundation Hospital South, Urbana, IL 61801 USA; 5 Carle-Illinois College of Medicine, Urbana, IL 61801 USA; 6 Cancer Center at Illinois, Urbana, IL 61801 USA; 7 Biomedical Research Facility, 3rd Floor Mills Breast Cancer Institute, Carle Foundation Hospital South, Urbana, IL 61801 USA

**Keywords:** Cancer, Carcinoma, Gastrointestinal, Stomach, Esophagus, Pancreas, Bile duct, Nanoparticle, Polymer, Liposome, Drug delivery, Treatment

## Abstract

Upper gastrointestinal (GI) carcinomas are characterized as one of the deadliest cancer types with the highest recurrence rates. Their treatment is challenging due to late diagnosis, early metastasis formation, resistance to systemic therapy and complicated surgeries performed in poorly accessible locations. Current cancer medication face deficiencies such as high toxicity and systemic side-effects due to the non-specific distribution of the drug agent. Nanomedicine has the potential to offer sophisticated therapeutic possibilities through adjusted delivery systems. This review aims to provide an overview of novel approaches and perspectives on nanoparticle (NP) drug delivery systems for gastrointestinal carcinomas. Present regimen for the treatment of upper GI carcinomas are described prior to detailing various NP drug delivery formulations and their current and potential role in GI cancer theranostics with a specific emphasis on targeted nanodelivery systems. To date, only a handful of NP systems have met the standard of care requirements for GI carcinoma patients. However, an increasing number of studies provide evidence supporting NP-based diagnostic and therapeutic tools. Future development and strategic use of NP-based drug formulations will be a hallmark in the treatment of various cancers. This article seeks to highlight the exciting potential of novel NPs for targeted cancer therapy in GI carcinomas and thus provide motivation for further research in this field.

## Introduction

The upper digestive tract refers to a system of organs that includes, but is not limited to, the esophagus, stomach, bile duct, gallbladder and pancreas. Upper gastrointestinal (GI) cancer is one of the leading causes of cancer death worldwide [1]. In 2017, more than 864,989 patients died from stomach cancer, 441,083 from pancreatic cancer, and 173,974 from biliary system related cancer with numbers increasing each year [1]. Moreover, the diagnosis and treatment of malignant tumors within the GI tract remain both challenging and problematic. Notably, GI cancer is often clinically silent in its development until the symptomatic discovery reveals that the disease has already progressed to an advanced stage. Additionally, GI oncogenesis forms micrometastasis in early developmental stages. Therefore, only 10% of patients with pancreatic cancer and 10–20% with gastric cancer are suitable for surgery at the time of diagnosis [2]. This leads to high local and systemic recurrence rates within 5 years after the surgical removal of solid tumors with values reaching as high as 80–90% for pancreatic cancer and 60–70% for stomach and bile duct cancer [3, 4]. Furthermore, the development of a dense capsule comprised of connective tissue that covers the core of the solid tumor constitutes yet another substantial hurdle in the treatment of GI carcinomas. The opaque stroma widely impedes drug penetration and protects the cancerous cells from chemotherapy. Advances described below focus on developing drug delivery methods capable of passing through the fibrous barrier to deliver therapeutic agents at targeted sites, ultimately minimizing toxicity.

Integrating the challenges related to diagnosis, aggressive metastasis, impeded drug penetration, and high recurrence rates marks GI cancers as some of the deadliest tumor types. Local and distant recurrences remain the rule rather than the exception, suggesting that cancer cells remain present, even after aggressive surgical removal of gross evidence of the disease. Ultimately, therapeutic advancements will identify and address the unique biochemical, physiological and genetic processes which promote the lethality of this disease. Indeed, a one-treatment cure seems unlikely, rather, improved survival may more likely result as multiple agents and modalities address particular difficulties. For example, in colorectal cancers, survival rates have improved as effective targeted therapy, immunotherapy, cytotoxic chemotherapy and radiation therapy more effectively control microscopic and locally invasive disease, allowing for durable disease-free intervals even in advanced cases. However, during the same period, novel treatments for upper GI cancers have led to only limited improvements in survival. Thus, emerging therapies should pursue the incorporation of intelligent tools that minimize toxicity and unwanted side effects via targeted approaches to a given tumor, with future success resting in the cooperative and additive application of innovative multimodality treatments.

### Current therapy of pancreatic cancer

Pancreatic cancer (PC) accounts for approximately 7% of all cancer deaths worldwide and 3% for cancer deaths within the United States [5], making it the $5{\text{th}}$ most common cancer-related death in the United States and the $4{\text{th}}$ most common cause of cancer-related deaths in Europe [6]. The average 5-year relative survival rate for all localization stages of pancreatic cancer is 5–9% [7]. When detected in its early stages, the surgical removal of the cancerous tissue is often the most promising treatment available. Neoadjuvant (given before surgery with the intent to cure) chemotherapy is administered to improve the likelihood of complete tumor removal and mitigate micrometastatic growth. Adjuvant chemotherapy (administered after surgery) is given in hopes of controlling micrometastatic disease. Radiation therapy may be applied to control the disease locally before or after surgery. Palliative chemotherapy is provided to patients with inoperable disease in an attempt to control symptoms and delay death. Current chemotherapy treatments often entail combined regimens such as FOLFIRINOX (5-fluorouracil [5-FU], leucovorin, irinotecan, oxaliplatin) which demonstrated improved post-surgical survival [8]. Other chemotherapeutic drugs include $capecitabine^{TM}$, $paclitaxel^{TM}$ (PAX), $erlotinib^{TM}$ and $gemcitabine^{TM}$(GEM). In 2019, the polymerase inhibitor $olaparib^{TM}$ was approved by the U.S. Food and Drug Administration (FDA) for metastatic PC [9].

Though some of these newer agents and regimens have improved survival, this has been on the order of only a few months and progression of the disease occurs in more than 90% of patients. Thus, it is necessary to explore alternative treatments such as NP-based therapies that are able to more effectively target and treat complex cancerous tissues. Targeted therapies are in development which could address the unique challenges presented by pancreatic adenocarcinoma (PA). Novel agents are being designed which modify various processes thought to be responsible for PA virulence. The targeted mechanisms include signaling pathways such as the ones mediated through phosphoinositide 3-kinases (PIK3), supportive processes such as angiogenesis and the desmoplastic environment, immune response augmentation, and agents which may work towards interrupting the epithelial to mesenchymal transition.

To date, only a handful of NP-based therapies have been approved for clinical use. One such example is the nanoliposomal irinotecan, a topoisomerase inhibitor, which acts as a second line of treatment following GEM-based chemotherapy for advanced PC [10–13]. Furthermore, NP-bound albumin with encapsulated PAX may be used in combination with GEM and has been approved as a standard therapy with evidence of increased tumor stroma depletion [14]. These NP-based treatments demonstrate promise as further research is performed within the field of NP-based cancer therapies.

### Current treatment of bile duct carcinomas

Cholangiocarcinoma is a malignancy originating from the biliary epithelium and develops anywhere along the biliary tree. The overall prognosis for cholangiocarcinoma remains poor with a 5-year survival rate ranging between 8 and 24% [7], depending on the stage of the disease at the time of discovery. Similar to PC, the most effective treatment for advanced bile duct carcinomas (BDC) is surgery, particularly surgical resection. Despite chemoresistance present in BDC, the ABC-02 trial confirmed that GEM in combination with cisplatin could be used for advanced unresectable BDC and was effectively able to prolong patient survival by an average of 4 months [15]. Further treatment options for biliary tract cancers include adjuvant combination therapy performed for at least 6 months with capicitabine, FU-5, paclitaxel or irinotecan. Moreover, the use of nab-PAX and GEM have emerged as the standard chemo-agents in co-formulating therapeutics for front-line treatments. Neoadjuvant application of the substances was shown to increase treatment success and survival after surgery in BDC [16]. In 2020, the FDA approved $pemigatinib^{TM}$, which targets the hyperactivity of the oncogenetic fibroblast growth factor receptor 2 (FGFR2)—for unresectable advanced BDC [17]. Current standard of care plans may include chemotherapy; however, surgery remains the primary treatment for a non-metastatic disease. Depending on the location of the tumor, intraductal ablation therapy can be administered by placing a radioactive probe at or near the tumor site. Meanwhile, unresectable and/or metastasized BDC are treated with external radiation therapy. A uniform treatment strategy remains to be developed for adjuvant and neoadjuvant therapies. Due to intrinsic drug resistances, research on novel agents and drug combinations is fundamental for the cure of BDC.

### Current treatment of gastric and esophageal cancer

Gastro-esophageal cancer (GC) is the third leading cause of cancer-related deaths worldwide [18] and is characterized by malignant tumors that decrease the 5-year survival rate of patients to less than 20% [7]. However, the survival rate is strongly dependent on the stage at which the disease is diagnosed with an improved outlook accompanying early diagnosis. Currently, the primary treatment for early stage Gastro-esophageal cancer is based on a combination of neoadjuvant chemotherapy or chemoradiotherapy.

Pre-surgical treatment for non-metastatic gastric and esophageal cancer is performed to optimize therapy by controlling the micrometastasis, decrease the size of the tumor and test its aggressiveness. These actions allow treatment tailored to patient-specific needs, as well as mitigate post-operative complications while increasing the likelihood of complete tumor removal. Though surgical interventions have improved, the parallel application of chemotherapy or radiochemotherapy provides better clinical outcomes. Gastric cancer treatments employ a recently developed neoadjuvant chemotherapy regimen consisting of synergetic medications using the FLOT scheme: docetaxel $60 \frac{mg}{m^2}$, infusional 5-fluorouracil $2600 \frac{mg}{m^2}$, leucovorin $200 \frac{mg}{m^2}$, oxaliplatin $85 \frac{mg}{m^2}$. This method has shown improved survival rates compared to previous regimens. For esophageal cancer, PAX and carboplatin are common pre-operative regimens while fluorouracil or capecitabine are used post-operation per National Comprehensive Cancer Network (NCCN) guidelines. For patients with metastatic diseases whose tumors harbor particular genetic profiles, such as the over-expression of the human epidermal growth factor receptor-2 (HER-2) or neurotrophic tropomyosin receptor kinase (NTRK)- gene fusions, microsatellite instability, mismatched repair genes or programmed cell death ligand 1 (PD-L1) over-expression, targeted therapy or immunotherapy may be beneficial. Recently, $pembrolizumab^{TM}$ and $ogivri^{TM}$ targeting those genetic traits have been approved by FDA for the treatment of advanced GC [19, 20].

Meanwhile, surgical prospects such as minimally invasive esophageal resection has led to decreased postoperative morbidity [21]. For unresectable disease, treatment procedures with cisplatin and cebox prolongs survival. Despite significant improvements in the overall survival of patients made in recent decades, recurrence is common, and advanced stage disease remains incurable with a mean survival of 8–12 months. Nanomedicine offers the means to prolong patient viability by using nanoparticle delivery of conjugates such as nab-palitaxel, cisplatin, and capecitabine that are currently being tested in the neoadjuvant setting in a phase 2 clinical trial [22].

## Nanodelivery

**Fig. 1:**
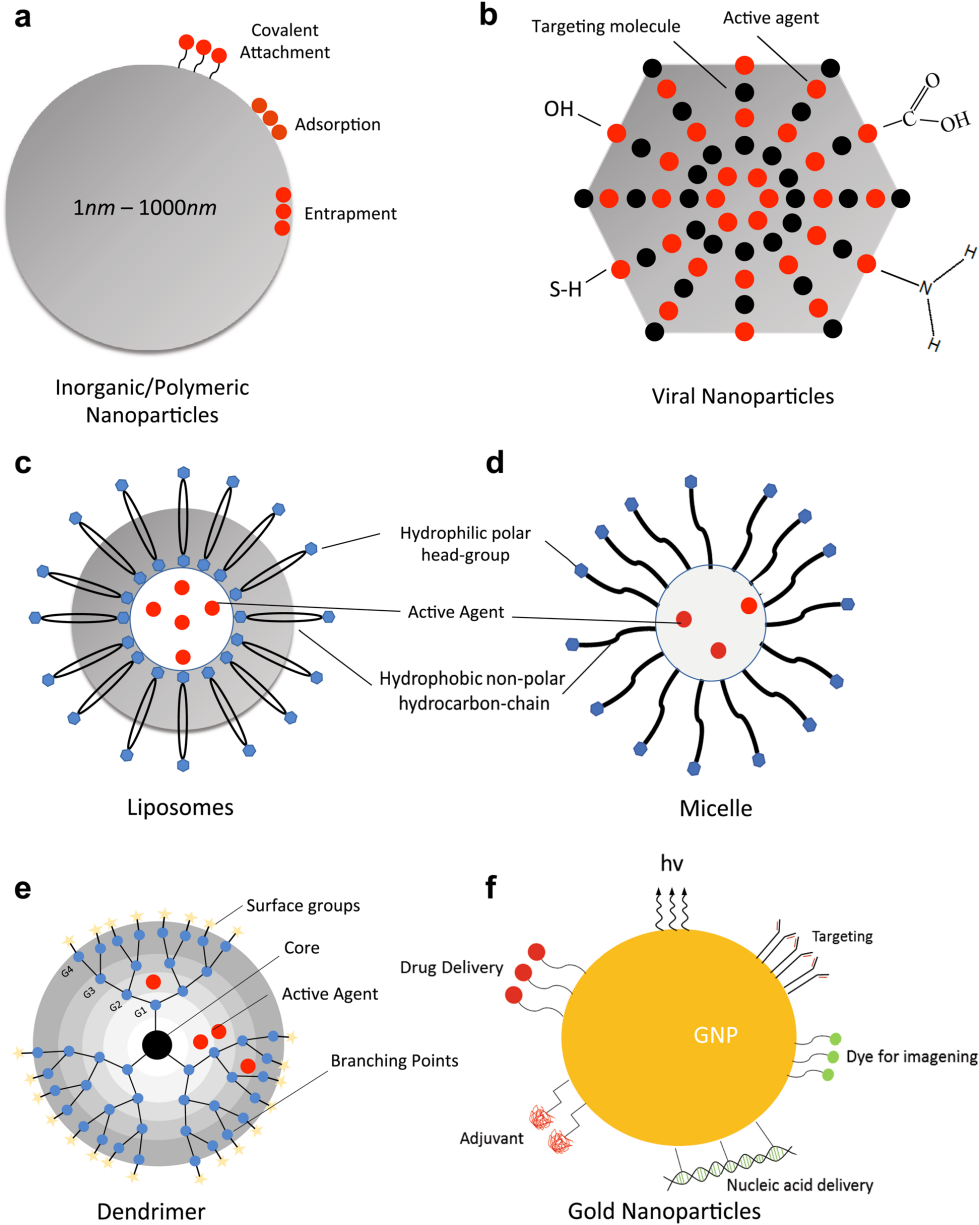
Nanoparticle features

Conventional chemo-treatment of cancer is impeded by limited circulation time, low concentrations within the intended treatment area, reduced water solubility, and toxic side effects due to disparate biodistribution, all of which reduce the overall efficacy of the treatment, as well as negatively impact patient morale. Therefore, drug systems operating at nanoscales have emerged as an improved pharmacokinetic approach to overcoming the deficiencies of current combination therapies. Nanoparticles (NP) are colloidal carriers varying between 1 and 1000 nm in size with natural or synthetic origins. The primary advantages of using NPs in the delivery of cancer therapy drugs are high specificity, increased efficiency, excellent stability and low overall toxicity for the patient [23]. There is a wide variety of nanocarriers available for drug delivery, including metal and polymer-based NPs, as well as liposomes. Types of nanocarriers and their characteristics are summarized in Fig. 1. The following sections review the trends in nanodelivery methods.

### Metallic & inorganic particles

Metal nanoparticles have gained considerable attention due to their promising properties and therapeutic applications in cancer treatment. Materials such as gold or iron oxides have revolutionized the approach to cancer therapy. Gold nanoparticles (GNPs) are established nanostructures that strongly absorb light, allowing them to generate thermal energy often resulting in the photothermal destruction of cancerous tissue [24]. Photothermal ablation occurs when GNPs are excited with a wavelength that corresponds to the specific surface plasmon resonance of the particle [25]. This is particularly desirable in GI carcinomas since surgical removal of these tumors is either too complicated or not possible for the majority of patients. Furthermore, GNPs are biocompatible and are considered non-toxic, making them desirable candidates for the targeted delivery of therapeutic chemicals [26]. At present, combination strategies involving both photothermal therapy and chemotherapy are used to increase the response of PC to chemoactive drugs [27]. This synergistic approach shows potent anti-tumor activity. Photothermal ablation is achieved via GNPs, selenium NPs, or copper sulfide NPs [28–30]. Another recent study demonstrated the application of GNPs when adhered to the surface of PAX loaded poly(lactic-*co*-glycolic acid)(PLGA)-microspheres [31]. Further research describes conjugated poly(ethylene glycol)-polylactide (PEG-PLA)-GNPs with doxorubicin (DOX), a tyrosine kinase inhibitor, and varlitinib, an anthracycline [32]. Upon radiation with near infrared light, the drug system presented an elevated cytotoxic effect on a PC cell line when compared to the effects seen without plasmonic activation of GNPs.

Additionally, iron oxides such as magnetite ($Fe_3O_4$) exhibit magnetic properties that can be exploited for guidance in enhanced tumor thermotherapy [33]. One major concern when using this material is the release of toxic hydroxyl radicals. Therefore, chemically functionalized maghemite ($\gamma{\text{-}}Fe_2O_3$) is used as an alternative in drug delivery systems targeting tumor cells via material surface modifications [34]. Recently, iron oxides have been conjugated with liposomes to create magnetoliposomes that are able to deliver chemical agents via selective drug release when exposed in a magnetic field [35]. Endeavors to create versatile and reliable delivery systems have led to inorganic carriers composed of carbon or silica-conjugates such as siliciumdioxide. For example, mesoporous silica NPs have been proposed as efficient delivery structures for therapeutic agents with peculiarities comprising of increased drug solubility, high loading capability, multifunctionality as well as stimuli-responsive release control. Among various beneficial characteristics, silica NPs are especially compelling for GI cancer applications since their surface can be easily modified to penetrate through GI barriers or target cancerous cells [36, 37]. These studies show tremendous promise in ongoing efforts to create reliable targeted delivery systems.

Yet another inorganic NP delivery system is the metal organic framework (MOF), which is constructed to perform a variety of functions [38]. MOFs are hybrid nanomotifs built from metal ions embedded in a network of organic linkers and recent evidence indicates that they are effective with respect to photodynamic therapy, as well as enhanced immunotherapy [39, 40]. Moreover, MOFs have a high drug-loading ability, biocompatibility, and multifunctionality. Evidence of this is provided through the application of stimuli-responsive MOFs with various functionalized coatings for programmable nanodelivery [41]. The study explored different MOFs, including zeolitic imidazolate frameworks-8 (ZIF-8), the UiO-66 (Universitetet i Oslo) framework, and MIL-101 (Matérial Institut Lavoisier), a combination of terephtalic acid and chromium salts. The NPs were coated with polydopamine (PDA), an oxidation product of catecholamines, which has excellent photothermal transduction properties. Conjugation of chemotherapeutics with PDA results in better solubility, as well as a more controlled drug release providing the opportunity to target tumor cells via chemo-photothermal destruction [41]. Additional research has been conducted linking UiO-66 MOFs to cytosine-phosphate-guanosine (CpG) as cancer vaccine adjuvants [42] while folic acid and HER2-based therapies have also been explored as a proof of concept with nanoparticles [43, 44].

Inorganic NPs, such as the ones described above may offer advantages over polymeric particles with respect to size and shape control, as well as the simplified preparation and functionalization of NPs. Additionally, these particles may be tracked more easily via magnetic resonance imaging (MRI), analytical techniques such as mass spectrometry, or optical techniques performed at the cellular level [45]. Yet, inorganic NPs may also have disadvantages related to degradability, elimination through the kidneys and oxygen radical formation leading to potential toxicity.

### Viral nanoparticles

Viruses may also be used to develop technologies in the fields of biomedicine and nanoparticles, particularly in tissue targeting and drug delivery. Viral nanoparticles (VNPs) are robust in their protection of nucleic acids due to the stability of the capsid. This increases the resistance of VNPs to both temperature and pH levels while allowing the VNPs to remain stable in a variety of solvents. Additionally, VNPs are often considered symmetrical, polyvalent, and monodisperse. Numerous VNP platforms have been developed, including icosaherdral plant viruses and bacteriophages, as well as rod-shaped plant viruses and filamentous phages [46]. The adaptability of these platforms allows modifications to be made such that binding of drug molecules can occur via encapsulation, adsorption, or covalent attachment.

Cargo molecules may be encapsulated through interactions with the interior architecture of the capsid, whose highly symmetric and repetitive surface provides multiple sites for the covalent attachment of site-specific residues [47]. Therapeutic drugs and imaging agents can then be chemically attached to reactive functional groups such as thiol for specific delivery [48–50]. The efficacy of this method has been validated through the use of a virus-like protein cage architecture used to attach and release the anticancer drug DOX [51]. This approach of drug delivery is advantageous because the protein cage protects the therapeutic compound from the exterior environment, making it inert until the correct time and location for drug delivery is reached.

Viruses may also present molecules exposed on their surface that allow for host recognition, implying that the virus may avoid host defense mechanisms. Additionally, viruses can also have an affinity for receptors of biomolecules that are up-regulated in tumor cells [52]. To combine these two desirable qualities, ligands may be attached to the exterior of the viral surface, incorporating a variety of antibodies, targeting molecules, and peptides onto the surface of the capsid using chemical or genetic means of adhesion [53, 54]. Such an approach has been shown to provide cell-specific and tissue-specific targeting with applications in the delivery of therapeutics or imaging agents [55, 56]. In vivo tumor targeting has utilized antibodies such as transferrin, folic acid, and single-chain antibodies [57]. Similarly, thiol groups may be exposed on the surface allowing for viral capsid monolayers to be patterned on gold surfaces [58]. Furthermore, amino acid residues are used to modify the protein cage architecture so that the reactive sites are available to bind small molecules for site-specific attachments on gold nanoparticles [59], fluorophores [48, 50], carbohydrates [60], nucleic acids [61], and peptides [55, 56]. Additional VNP platforms and their applications in drug delivery and imaging are reviewed in prior work [47, 57, 62–68]. Further research on synthetic compounds may provide a broader range of possible small molecules that could be attached for delivery via virus-based NPs [69, 70].

### Polymers

Polymers are large molecules consisting of recurring structural units (known as monomers) that are joined with covalent bonds and can also be utilized as a vehicle for drug delivery to treat cancer. Polymers have several beneficial qualities with regard to drug delivery, patient safety and manufacturing.

During preparation of polymer-based systems for drug delivery, the drug is either entrapped or covalently bound to the matrix of the polymer [71]. Thus, there are multiple resultant structures such as polymeric NPs, micelles, or dendrimers [53]. Schematic representations of some of these systems are shown in Fig. 1. Polymers used in drug delivery systems can be divided into two categories: natural and synthetic. Example compounds for each polymeric system of drug delivery in GI cancers are provided in Table 2.

#### Polymeric nanoparticles

Polymeric nanoparticles are defined as colloidal carriers that must be less than 1 $\upmu$m in size [72]. When selecting a polymer for the formation of a NP for drug delivery, several design factors must be considered, including the desired size of the nanoparticle, its surface characteristics, the biodegradability and biocompatibility of the polymer being used, as well as features of the drug being delivered, such as solubility, stability, carrier method, and drug release profile. Moreover, the polymers utilized must also be characterized as non-toxic, non-thrombogenic, non-immunogenic, and non-inflammatory. Potential polymers should also avoid the activation of neutrophils, induction of platelet aggregation, and the reticuloendothelial system while having prolonged circulation time, an adequate elimination profile, as well as being cost effective and scalable [72].

Natural polymers are ideal candidates for the delivery of drugs due to their ability to enhance drug availability to the target tissue [73]. Additionally, it is characterized as a safe means of delivery due to its biodegradability and lack of toxicity. Natural polymers such as polysaccharides (e.g. chitosan), cellulose, alginate (ALG), amino acids, and proteins (e.g. gelatin and albumin) are commonly chosen materials for delivering DNA, proteins, and other drugs to targeted tissues [73–75]. Based on their physio-chemical properties and biocompatability, biopolymers have gained increasing attention in targeted cancer therapy. Amongst these, natural cellulose and chitosan have advantageous properties. Due to the high stability, workability, specific surface area as well as pH-responsiveness, cellulose and chitosan have become attractive matrices for drug delivery systems. Both polymers are approved as safe by FDA. Hence, current studies and clinical trials explore the potential of these materials loaded with chemodrugs such as 5-FU, PAX, DOX and the promising natural anti-inflammatory compund Curcumin. These drug platforms are accelerating progress towards novel therapeutic compounds in GI carcinoma favoring their muco-adhesiveness and ph-sensitivity. A summary of functions and applications of cellulose and chitosan NPs can be found in recent excellent reviews [76, 77]. Ample opportunities exist in creating nano-delivery systems with site-specific targeting capabilities to increase the efficacy of therapy and minimize toxicity.

Synthetic NPs are prepared from polymeric materials such as poly(ethyle-nimine) (PEI) [78], poly(alkyl cyanoacrylate) (PACA) [79], poly($\eta$-caprolactone) (PCL) [80], PLGA [81–83], or (poly(lactic acid) (PLA) [84]. These carriers can transport drugs in a variety of ways [85]. Other classes of NPs have been designed to deliver adjuvants such as oxygen to mitigate cancer hypoxia to increase the effectiveness of successive therapies [86–88]. Despite their advantages, the application of most polymeric NPs is still immature due to widely unknown cellular interactions, low solubility in non-acidic milieu and poorly conceived methods for large scale production.

### Polymeric micelles

The second polymer-based drug delivery system are polymeric micelles, whose functional properties are based on amphiphilic block copolymers [53]. When assembled, these copolymers form a nano-sized shell within an aqueous solution. The hydrophobic core serves as a reserve for the hydrophobic drug being delivered. Drugs can be loaded into the polymeric micelle in two ways: encapsulation [89] or covalent attachment [90]. Thus, the polymeric structure is water-soluble and can be delivered to a patient through intravenous administration [91].

Only few micelles for cancer therapy have reached clinical practice. Presently, polymeric micelle delivery systems are faced with drawbacks such as non-targeted delivery, difficulties in creating multidrug systems and reduced drug resistances. However, micellar drug formulations have potential for future clinical use with emergence of one of the most successful nanocarriers: PEG–PLA micelles [92]. Functionalized PEG-PLA micelles are capable of delivering chemotherapeutics, photothermal and photodynamic therapy components, tumor-associated antigens, nucleic acids, and anti-tumor agents such curcumin or doxorubicin during antivascular therapy [93]. Current clinical trials are testing multiple untargeted PEG-PLA micelles containing various drug compositions [94]. Further developments should aim to overcome drug resistances and provide active tumor targeting, and combine multiple active agents. In efforts to pursue these goals, arginine-glycine-aspartic acid (RGD)-decorated PEG-PAX conjugates have been designed for the treatment of gastric cancer [95]. These conjugates assemble autonomously into micelles that are able to release PAX in weakly acidic environments. Further toxicity studies must be performed to integrate micelles into approved treatment regimens.

#### Dendrimers

Dendrimers are synthetic polymeric macromolecules, composed of branched monomers that radially extrude from a central core. Their structure allows for a more modifiable surface functionalization, increased solubility, and an internal cavity for drug delivery [96]. This permits the conjugation of several molecules such as contrast agents, targeting ligands, nucleic acids, or multiple therapeutic drugs. One such dendrimer that has gained attention in the medical field is the poly(amidoamine) (PAMAM) dendrimer. This platform has been growing in popularity due to its multiple functionalities, biocompatibility, and the ability to adjust its size [97], making it an ideal candidate for drug and gene delivery, medical imaging, and sensing [98–103].

Presently, researchers are exploring the concept of dendrimers as radiopharmaceuticals, which combine radiotherapy and gene or antibody delivery [104]. These approaches could enable more efficient targeting and reduce the dose of radioactive materials. Furthermore, the potent anti-cancer drug PAX has limited applications due to low hydrophilicity and permeability, which decreases NP-conjugation. PAX-conjugated PAMAM demonstrates a significantly higher cellular uptake compared to polymeric carriers [105]. Moreover, superparamagnetic iron oxide or gadolium dendrimers have emerged as a new class of nanovehicle for cancer theranostics when applied in conjunction with MRI techniques [106]. These structures combine MRI detection with controlled drug release by modulating pH, temperature, or inducing magnetic hyperthermia. Additionally, these nanostructures are exploited in MRI scans for their properties as contrast agents, as well as their response to the magnetic field gradient used to guide NPs to tumorous sites. Thus, dendrimers have proven to be efficient nano-architectures for diagnosis, imaging, and therapy. Despite their current in vitro success, further developments of dendrimers should focus on increasing bioavailability and degradability while reducing the intrinsic toxicity of anti-cancer agents.

### Liposomes

Liposomes are closed colloidal structures capable of self-assembly. Their structure is composed of lipid bilayers that produce a spherical shape surrounding an aqueous center. Thus, it is possible to encapsulate both hydrophilic and lipophilic drugs [107, 108]. Solid lipid NPs have proven to be efficient carrier systems due to their ability to release therapeutic agents directly into the cytoplasm [109]. The specificity of this treatment can be further improved through the use of targeting deliver the necessary therapeutics. Cancerous tissues often have leaks within the vasculature [110], allowing liposomes to accumulate in a way that non-NP-based drugs cannot. This leads to enhanced retention and increases the concentration of the drug within the targeted tissue [107, 111, 112]. Moreover, liposomes may also be coated to reduce interactions with components within the blood, which, in turn, reduces the likelihood of the nanoparticle being retained in the reticuloendothelial system [113]. This shielding mechanism extends circulation time and allows for longer exposure in the affected tissue regions [114]. Furthermore, active targeting using liposomes is achieved by coupling targeting ligands to the surface of a liposome through either covalent or non-covalent bonds. This allows liposomes to be selectively paired with cells that overexpress the receptor for the ligands [115, 116]. Thus, the efficacy of the drug delivery system can be increased while the off-target toxicity of treatment can be mitigated to a significant extent. This can further be extended to functionalizing the surface with antibodies to produce immunoliposomes. Although not discussed in detail within this article, a review of immunoliposomes and their functionalization was conducted [113].

Despite the advantages of liposomes, poor stability and reduced availability during oral administration are considered general limitations of the method. Recent work has sought to increase drug adsorption through surface modifications such that liposomal nanocarriers could withstand the harsh acidic conditions in the GI tract [117]. Meanwhile, in vitro studies have discovered that silica-coated flexible liposomes or N-trimethyl chitosan chloride-coated liposomes increase the absorption of Curcumin by improving particle stability and water solubility [118, 119]. Moreover, enhancing drug bioavailability, longevity and target specificity has been evaluated using liposomes in nanohybrid delivery systems with conjugates like GNPs or dendrimers [120, 121]. Adding to previously approved liposomal PAX, nanoliposomal cisplatin and DOX are currently used in clinical trials for pancreatic and other GI cancers [122, 123]. Lipid-based drug delivery systems provide a promising outlook on future treatment of cancer with current research exploring NP-drug combinations that highlight the advantages of NPs while mitigating the disadvantages associated with the emerging technology.

## NP-based GI carcinoma therapies

Given the advantages of tailored drug delivery, targeted NPs has the potential to be more effective and a lesser toxic treatment option. Not only do these drug delivery systems have prolonged circulation and protection against premature drug degradation, they also facilitate drug penetration, targeted delivery, and an increased concentration of the drug in the specified cancerous tissues. NPs provide an innovative platform for drug delivery since the structures allow for a range of sizes, shapes, chemistries, and surface charges. By modifying these elements, there is improved control over their stability of the drug, as well as the timing and location at which the therapeutics are released. A summary of key NPs in targeted cancer nanomedicine can be found in Table 2, whereas Table 1 provides an overview of specific applications of various NPs in GI carcinoma therapy.

**Table 1:** Key NPs in GI cancer therapeutics

Composition	Material	Therapeutic agent	Target	Model	Refs.
Inorganic	Silica NP	IDO-inhibitor + oxaliplatin	P	Transgenic Pdx-A-Cre mouse	[164]
		PAX + gemcitabine	P	PANC-A mouse xenograft	[154]
		PAX + curcumin	GC, BD, P	4T1 mouse xenograft	[165]
		Irinotecan	P	Kras-derived PDAC mouse model	[166]
	Selenium	Oridonin + GE11 peptide	GC	Human esophageal cancer cell lines (KYSE-150 and EC9706) and KYSE-150 xenograft mouse model	[167]
Metallic	Gold	Doxorubicin + VARLITINIB	P	Cancer line S2-013s	[32]
		HER-2 siRNA	GC	MFC-derived tumors bearing mice	[152]
Natural	Albumin	PAX + gemcitabine	BD/P/G	Applied in Clinics	[168]
		Hyaluronic acid coated, load: celastrol + A-Methyl-Tryptophan	P	C57BL/6 mice xenograft model	[141]
	Chitosan-PLGA	Docetaxel + elacridar	P, GC	A549 adenocarcinoma cell culture	[169]
	Alginate	Liquid alginate	GC	Clinical trial for pre-cancerous Barrett’s esophagus	[170]
Polymeric	PLGA	PAX-oncoGel	P	Porcine pancreas in vivo, phase I clinical trial	[171]
		Docetaxel + LY294002	GC	Orthotopic GC—and xenograft mouse model	[172]
		5-fluoroucil + PAX	GC	In vitro and Ex vivo sLeA cells	[173]
Liposome	Phospholipid	IDO-inhibitor + oxaliplatin	P	Syngeneic mice	[164]

*P* pancreas, *BD* bile duct, *GC* gastric/esophageal

**Table 2:** NP materials in targeted cancer therapy with potential in GI treatment

NP Type	Material	Advantages	Disadvantage	FDA approval/clinical status	Refs.
Polymer	PLGA	Enhanced drug solubility,improved accumulation at tumor site, completely biodegradable, non-toxic clearance, suitable for surface modifications, dual functionality when conjugated with chitosan or gold, use in photothermal therapy, vaccine and gene delivery, easy and proven processing	More toxicity studies and clinical evaluation needed, possible immunogenic properties	Approved for drug delivery. Clinical trial: PEG-PLGA docetaxel, paclitaxel PEG-PLGA, paclitaxel oncoGel	[174, 175]
	Chitosan	Antimicrobial properties, solubility, stability and biocompatability, coating for other NPs, cationic and mucoadhesive characteristics—ideal for oral delivery, permeation enhancement, pH responsive, excellent gene and cancer vaccine delivery vector	Low solubility in non-acidic pH, lack of comprehensive toxicity profile, deacetylation degree determines physio-chemical behaviour, crosslinking might prevent degradation	Approved as safe. Animal studies on DOX-chitosan. Clinical studies (mainly lung and breast cancer): chitosan NPs loaded with curcumin, cisplatin or ascorbate	[77, 176]
	Collagen	Conjugation with metal NPs, high biocompatibility, suitable for inhalation, controlled release	Delivery of collagenase desireable in cancer—not possible with collagen, difficult particle fabrication	Research: silver NP stabilized with collagen	[177, 178]
	Alginate	Mucoadhesive, pH sensitive, oral delivery, suitable for micelles, stabilizer for metal NPs	Unknown toxicity, need stabilizers	Preclinical studies: magnetic chitosan/alginate- curcumin NP, exemestane-ALG-NPs, paclitaxel-loaded ALG-NP	[155, 156]
	Cellulose	pH triggered release, completely biodegradable, oral administration, increase drug solubility	Difficult fabrication, aggregation due to hydrophylic nature, no release in acidic pH (GC), insufficient knowledge about interaction with cells and tissues	Approved: carboxymethyl cellulose. Clinical Trial: eethylcellulose + cetuximab	[76, 179, 180]
Metal	Se	Antioxidant, anti-inflammatory properties, anticancer activity, dual delivery of therapeutics like siRNA + cisplatin, curcumin SeNPs	Dual role in cancerogenesis and drug delivery—might also produce radicals, possible epigenetical modifications	Only in vitro cell studies	[167, 181, 182]
	Au	High surface to volume ratio, stable, ideal plasmon resonance for therapeutic applications like photothermal and photodynamic therapy, good biosafety profile, high permeability, scalable	Limitations regarding bioavailability of drugs, possible cytotoxicity depending on shape and size, changes in gene expression	Approval: DOX-AuNP, clinical trial: oxaliplatin-platinium-AuNP	[183]
	Superpara-magnetic $Fe_3O_4$	Magnetic nanocomposites as contrast agents for MRI imaging, drug release control, hyperthermia agents, enhancement of radiation therapy	Possible immunotoxicity, toxicity due to formulation of hydroxyl radicals	Approval only for ferumoxytol (chronic kidney disease), to date no FDA approval for cancer imaging and treatment, late stage clinical trials for Nanotherm(R) ablation therapy (currently approved in Europe for Glioblastoma)	[33, 34, 184–186]
Inorganic	$SiO_2$ carriers	Large specific surface area and pore volume for drug loading, controlled release kinetics, targeted delivery via surface modifications, endocytotic behavior, good biocompatibility, suitable for oral administration and bioimaging	Insufficient information about clearance time, immunogenicity and accumulation in tissue, lack of toxicity data	Silica NP with C dots approved for stage I clinical trial, in vitro cell studies for Paclitaxel-loaded SeNPs	[36, 37]
	Carbon NPs/nanotubes graphene oxide, nanodiamonds	Resist harsh acidic environment, biocompatible, able to overcome GI barriers, thermal conductivity, scalable	Poor bioavailability and solubility, intrinsic toxicity	HeLa cell studies for Cisplatin delivery	[163, 187, 188]
Lipid	Liposome	Excellent solubility, high bioavailability and biocompatibility, biodegradable, drug protection, thermosensitive, multidrug loading	Rapid clearance without stabilizing conjugates, possible toxicity, poor knowledge about internalization processes,	Approval: nanoliposomal Irinotecan, PEG-liposomal doxorubicin. Clinical Trial: paclitaxel liposome, liposomal doxorubicin, cisplatin, oxaliplatin, aroplatin and GEM	[122, 189–191]
	PEG-lipid micelles	Improved stability and drug solubility, enhanced permeability and retention due to small size ($<100\,\text {nm}$), cell internalization, programmable thermo-responsiveness, increased effectiveness against resistant cancer, multidrug loading	Antibody response against PEG component, lack of consistent behaviour regarding biodistribution and absorption	Approved: the polymer PEG. Clinical trials: Paclitaxel micelles in combination with cisplatin, docetaxel-polymeric micelles and oxaliplatin, gemcitabine with micellar cisplatin (NC-6004), curcumin-loaded micelles	[93, 192–194]
Protein-Based	Pyruvate dehydrogenase E2	Biomimetic platforms for mimicking, viruses, dendritic cell activation, and cross-presentation, cancer vaccine platform	B16 melanoma murine model	Only delay in tumor development, no clinical studies	[195]
	Albumin	High solubility of chemodrugs like paclitaxel, various binding capacities, excellent safety profile, biodegradable	Approved: Nab-Paclitaxel	Oral delivery not possible due to degradation in GI system, binding of active agents could lead to protein conformational changes	[196]

Chemotherapy is a prominent method of medication used in a significant portion of metastasized tumor treatments. However, the non-specificity of the treatment can cause serious toxic side effects and increase the resistance of the tumor to multiple drugs. Current research seeks to apply NPs in conjunction with an expanding knowledge of cancer development to more quickly and effectively address the diseases. Several studies have discovered that there is a correlation between chronic inflammation sites and tumor genesis [124, 125]. Consequently, present therapeutic strategies involving NPs have focused on developing anti-inflammatory elements, engineering anti-cancer immunity [126], as well as developing delivery platforms for co-formulating drugs [127]. Specifically, nano-encapsulation of Curcumin in liposomes and polymeric NPs has shown increased bioavailability that results in reduced inflammation and apoptotic effects for cancer cells [128, 129].

Whereas the opposite event of an immuno-suppressed tumor environment stimulates cancer angiogenesis. Other treatments seek to trigger the innate and adaptive immune system and boost its activity against cancer cells. Treatment procedures like T-cell based immunotherapeutic strategies are grounded in tumor-specific antigen delivery or the removal of inhibitory signals, resulting in the activation of T-cells [130]. Nanodelivery methods improve the clinical outcome of immunotherapies in a synergistic manner [131, 132]. When applied simultaneously with a chemo-agent, these processes were proven to be a highly potent form of medication [133].

Further research is being performed to determine how NPs might be used to disrupt the dense, fibrous stromas of GI tumors and their hypoxic microenvironments using hyaluronidase and/or collagenase [134–136]. Moreover, information about the molecular and genetic composition of tumors could be used to improve the safety and effectiveness of treatments in GI cancer, such as cisplatin, by providing targeted delivery of therapeutic genes or DNA repair enzymes [137]. In addition, multiple genetic markers enable differentiation between healthy and cancerous tissues and can, therefore, be used for selective nanodelivery of small interfering RNA (siRNA) or micro-RNA, which either silences or disrupts cancerous genes [138]. Subsequent sections discuss the present state of NP-based therapies with respect to various GI malignancies.

### NP-based therapies for pancreatic and bile duct cancer

Due to a limited number of treatment options with low treatment efficacy, PC remains one of the most fatal cancer, resulting in a patient 5-year survival rate of less than 5%. Therefore, significant emphasis has be placed on developing more targeted and efficient therapies. Nanosystems have demonstrated enhanced bioavailability by promoting prolonged drug circulation without degradation [139]. Moreover, certain NPs are able to increase the penetration rate of the drug into cancerous cells. Notably, collagenase NPs combined with PAX-micelles have been used in pancreatic ductal adenocarcinoma treatments to improve anti-tumor activity [136]. The proteolytic-enzyme complex encapsulated in a $100\,{\text{nm}}$ liposome cleaves excessive extracellular matrix and facilitates drug invasion. Meanwhile, PEG-PLGA NPs coated with neutrophil membranes were shown to overcome the blood-pancreas barrier and actively accumulate in cancerous tissue of a mouse model [140]. The bio-mimicking nanocarriers were loaded with celastrol, a pentacyclic triterpenoid extracted from *Tripterygium wilfordii*. Celastrol displays anti-inflammatory properties by inhibiting the activation of nuclear factor $\kappa$ light chain enhancer of activated B-cells (NF-$\kappa$B) and acts as an anti-cancerogenic agent, which locally reduced tumor size and inflammation.

Further enhancements in chemotherapeutics have been exhibited through the application of cationic albumin NPs loaded with hydrophobic celastrol and hydrophilic 1-methyltryptophan as a combination therapy with an indoleamine-2,3-dioxygenase (IDO) inhibitor [141]. Recent work describes the use of superparamagnetic iron oxide NPs (SPION) linked to Curcumin, a bioactive anti-cancer agent with promising effects in fighting malignant tumors [142]. Furthermore, chemoresistance to gemcitabine and tumor sphere formation were significantly reduced following the administration of Curcumin via SPION in an orthotopic mouse model [143]. GEM efficiency was also enhanced using the novel PAX nanoformulation in PLGA-NPs [144]. In this particular study, the lipid synthesis that leads to tumor survival and progression was inhibited by PAX delivered specifically to pancreatic cells. Similarly, GEM was delivered to pancreatic tumors with activatable liposomes [145]. These medical procedures are currently in clinical trials where the prevailing conclusions is that NP-based drug delivery methods are more effective in treating GC [146].

### NP-based therapies for gastric & esophageal cancer (GC)

As with other upper GI carcinomas, gastric cancer develops asymptomatically and is a significant cause of cancer-associated deaths. However, NPs can be effectively employed in the early detection of GI cancer. GNPs-RNA conjugates are used in the coloriometric detection of biomarker micro-RNA in GC [147]. In the treatment of advanced GC and its metastasis, evidence indicates the improved effectiveness of nab-PAX [148, 149]. These formulations could be used to increase the therapeutic value of the drugs by increasing the solubility. Interestingly, the binding of NPs to mucous tissues during oral administration sufficiently attenuated the progression of *Helicobacter pylori*-associated GC, which constitutes the root cause of approximately 60% of all GC [150]. Additionally, gene therapy consisting of exosomic NP-delivery of anti-mIR was used to reverse chemoresistance to cisplatin in GC [151]. As overexpression of HER-2 represents the major cause of GC development, the corresponding gene is an important target in GC therapy. While exploiting the photodynamic properties of gold, Zhang et al have suggested gold nanoshells for simultaneous delivery of HER-2-siRNA and photothermal cancer ablation [152]. Others demonstrated the delivery of gastric stem cell markers via specific antibodies loaded in PLGA-PEG-NPs in an attempt to create reliable target modalities [153]. As for other cancer types, there is ongoing research on the effect of curcumin against cancer cells in various animal models. Promising studies for GC include phospholipid-coated mesoporous silica NPs that have been simultaneously loaded with PAX and Curcumin to treat orthotopic mice models [154]. Alginate and cellulose have gained noteworthy attention for being mucoadhesive and therefore suitable canditates for gastric malignacies. There are various preclinical studies examining the effectiveness of PAX-ALG-NPs, Curcumin-alginate-NPs, ($Fe_3O_4$)-carboxymethyl cellulose NPs loaded with 5-FU on in vitro and in vivo GC models [155–157]. Fighting drug resistance, increasing the solubility and availability of the agents and reaching the GC tumorous cells in an effective manner remain major concerns in improving GC therapeutics.

### Future perspectives

NPs offer a promising route to deliver drugs that can be used in various stages of GI cancer. Over the past decade, nanotechnology has proven to be an important tool for the enhancement of novel chemo-immunotherapy for the treatment of various upper GI carcinoma. In future clinical practice, treatment of the tumor microenvironment, mitigating inflammation, and further activating the patient’s immune system will significantly affect the outcome of treatments. Presently, there is an urgent need for multifunctional tools capable of supporting and stimulating the immune response. Future efforts could focus on engineering anti-cancer immunity to minimize the toxic effects of chemotherapy drugs. While studies have identified multiple mutations, such as the oncogene K-rat sarcoma (KRAS), that are common in foregut cancers, attempts to interfere at the point of the gene product have not produced effective therapy. This has led researchers to increasingly focus on down stream molecular targets. Multiple such targets are now increasingly identified within the tumoral environment, including the stroma, angiogenic milieu and immune system. Other investigations have led to novel combinations of traditional cytotoxic drugs and have shown some promise. Given the multiple aberrant, virulence producing mechanisms that make foregut cancer among the most lethal, a single agent cure seems unlikely. Future inroads are best anticipated via mutimodality and multi-pathway interventions.

Several polymeric NPs have been used to simultaneously deliver tumor antigens and cell-specific adjuvants resulting in an effective stimulation of the immune system [158]. Meanwhile, other studies have focused on manipulating dendritic cells through PLGA-based NPs or GNPs to initiate an immune response against tumor cells [159, 160]. Recently, nano-vaccines have been suggested to normalize the microenvironment and relieve immunosuppression by delivering antibodies and recruiting T-cells into cancerous tissue [161, 162]. Oral uptake of chemo agents such as paclitaxel is decreased by multiple gastrointestinal barriers such as mucus, acidic environments, and the epithelium. Nanocarriers comprised of mesoporous silica or polymer-functionalized mesoporous carbon can be designed to overcome these obstacles while increasing the stability and solubility of the active agents [36, 163].

As the approach to cancer treatment shifts towards personalized medicine, individualized gene therapy could play a major role in the treatment of cancerous tumors with NPs delivering nucleic acids to targeted locations. Drug resistance is another obstacle that must be overcome to truly improve the survival outlook of patients with GI cancer. Since drug resistances are related to modified lipid biosynthesis, researchers are working on engineering effective NPs that can interact with lipid membranes to allow for enhanced uptake. However, these approaches still require further developments to efficiently target cancerous tissue. In conjunction, these novel strategies suggest an exciting potential for furthering the efforts of drug delivery systems used in the treatment of GI cancer.

## Conclusion

Pharmacokinetic shortcomings of current drug delivery techniques such as short retention time, non-targeted delivery, poor penetration into deep tumor tissues, lack of co-formulating agents, and high toxicity drive the development of novel drug delivery platforms such as nanoparticles. A wide variety of NPs are currently being researched with applications in chemotherapeutics, as well as photothermal therapy. The positive in vivo performance of nanomaterials relates to their unique properties that allow for controlled drug release, as well as both surface and cellular compatibility modifications. Presently, several NP-based systems, such as liposomes and protein-based NPs, are being applied in clinical practice for the treatment of GI carcinomas. These studies present evidence of an increase in patient survival. The use of NP-bound albumin loaded with paxlitaxel, nanoliposomal irinotecan as well as gold nanorods for photothermal ablation have been shown to be effective in targeting cancerous tissues and are applied in clinics at present. However, the field of nanomedicine still lacks standardization that would allow these therapies to become front line treatment options. Often, implementation of nano-based cancer therapies is impeded by uncontrolled toxicity and unforeseeable cellular interactions. Optimization of the structure and composition of NPs has yet to be performed to allow for a drug delivery system that is both non-toxic and programmable. This has proven to be a challenging proposition, as well as an opportunity for growth within the field. Furthermore, FDA approval rests upon novel drug formulations outperforming current treatment methods. Although several in vitro studies have been evaluated for the delivery of chemotherapeutics, clinical evaluations and deeper analyses of immunotherapies and combination therapies must be conducted before they can be adopted as standard treatment options.

## Data Availability

Not applicable.
